# Integrating Emotional Stress and Lipid Lowering in Cardiovascular Disease Management: The Future of Precision Cardiovascular Prevention

**DOI:** 10.3390/jcm14207208

**Published:** 2025-10-13

**Authors:** Emmanuel Eroume A Egom, Bernadette Sandrine Lema

**Affiliations:** 1 Heart and Vascular Institute, Department of Medicine, Hartford HealthCare, Hartford, CT 06106, USA; 2 Institut du Savoir Montfort (ISM), Ottawa, ON K1K 0T2, Canada; 3 Laboratory of Human Metabolism and Non-Communicable Diseases, Institute of Medical Research Medicinal Plants Studies (IMPM), Yaoundé, Cameroon; 4 Harmony Care, Heaven Foundation CIEL, Hartford, CT 06105, USA; lemabernadette@gmail.com

**Keywords:** cardiovascular disease, residual risk, lipid-lowering therapy, emotional stress, psychosocial factors, endothelial dysfunction, digital health, precision prevention

## Abstract

Residual cardiovascular risk remains substantial despite widespread adoption of intensive lipid-lowering strategies—statins, PCSK9 inhibitors, and RNA-based agents—that achieve very low LDL-C and apoB levels. Over the past three years, converging epidemiologic and mechanistic evidence has highlighted emotional stress—including anger, grief, anxiety, and chronic psychosocial strain—as a biologically active determinant of atherosclerotic disease and a frequent trigger of acute events. We propose the Emotion–Lipid Synergy Model, in which lipid burden establishes the atherothrombotic substrate while emotion-driven autonomic and vascular perturbations amplify endothelial dysfunction, microvascular constriction, inflammation, and thrombogenicity—thereby widening the residual-risk gap even when lipid targets are met. From this perspective, prevention should evolve toward precision psychocardiology: systematically screening for distress and stress reactivity; leveraging wearables to detect high-risk emotional states; and delivering timely, scalable, just-in-time behavioral interventions alongside guideline-directed lipid management. Particular attention is warranted for women and patients with angina and no obstructive coronary disease, who appear disproportionately susceptible to mental-stress ischemia. We outline a research agenda—flagship outcomes trials, mechanistic studies, and multimodal phenotyping—and discuss implementation pathways that integrate emotion metrics into cardiac rehabilitation and routine care. Integrating emotion assessment and modulation with lipid control offers a pragmatic route to reduce residual risk and advance equitable, personalized cardiovascular prevention.

## 1. Introduction

Despite major advances in lipid-lowering therapies—most notably statins, PCSK9 inhibitors, and newer RNA-based agents such as inclisiran—residual cardiovascular risk remains substantial. Even when low-density lipoprotein cholesterol (LDL-C) is reduced to ~30 mg/dL in large outcome trials, 9–10% of patients still experience major adverse cardiovascular events within just 2–3 years [1,2,3,4]. This highlights a persistent clinical gap that cannot be explained by LDL-C alone. Although additional strategies targeting lipoprotein(a), triglyceride-rich remnants, and apoB-containing particles are emerging, these approaches remain focused on lipid metabolism and do not fully address non-lipid drivers of risk [2,5,6,7].

Concurrently, emotional stress—including chronic psychosocial strain, anxiety, grief, and acute anger—has gained recognition as a biologically active determinant of cardiovascular disease. Stress activates the sympathetic nervous system, hypothalamic–pituitary–adrenal axis, and renin–angiotensin system, leading to endothelial dysfunction, oxidative stress, inflammation, and microvascular constriction [8]. Recent observational and mechanistic studies confirm that psychological distress independently predicts adverse outcomes in coronary disease, with particular vulnerability observed in younger women [9,10,11,12,13,14,15]. Taken together, these findings underscore the need to integrate emotional stress into prevention frameworks alongside lipid control.

It is important to distinguish between correlative and causal evidence when considering the role of emotional stress in ASCVD. Large observational studies and systematic reviews consistently demonstrate associations between psychosocial stress and increased cardiovascular risk, supporting its prognostic significance. However, randomized controlled trials provide more direct causal evidence. For example, structured interventions such as cognitive behavioral therapy (CBT), mindfulness-based stress reduction (MBSR), and supervised exercise programs have been shown to improve cardiovascular outcomes, functional capacity, and quality of life in patients with established heart disease [16,17,18]. These interventional findings reinforce the translational relevance of stress as a modifiable driver of residual cardiovascular risk, underscoring the clinical necessity of incorporating stress management into preventive frameworks.

Taken together, these findings point to an urgent need for integrative paradigms that address both lipid-driven and emotion-driven pathobiological pathways in cardiovascular disease. In response, we propose the Emotion–Lipid Synergy Model, a conceptual framework that situates emotional stress not as an ancillary variable, but as a central, co-equal determinant in the genesis and progression of atherosclerotic cardiovascular disease. This model underscores how emotional stress potentiates lipid-mediated injury—via inflammatory amplification, endothelial dysfunction, autonomic imbalance, and microvascular reactivity—creating a synergistic effect that magnifies residual risk even when lipid targets are met. What sets the Emotion–Lipid Synergy Model apart from existing biopsychosocial frameworks is its explicit focus on the biological crosstalk between lipid burden and stress-response pathways. Whereas traditional models treat psychosocial stress as a contextual factor, our model emphasizes its role as a *biological amplifier* of lipid-driven atherothrombosis—through neuroendocrine activation, NLRP3 inflammasome signaling, oxidative stress, and endothelial dysfunction [19,20,21,22,23,24]. The model is therefore designed with three complementary aims: (i) to provide a mechanistic framework that integrates lipid biology with emotional stress physiology; (ii) to enhance risk stratification by identifying subgroups whose lipid-related risk is magnified by stress reactivity; and (iii) to guide clinical intervention design, offering a conceptual scaffold for combining lipid-lowering therapies with stress-targeted strategies such as cognitive-behavioral interventions, mindfulness, or pharmacological modulation. By framing stress as a co-equal determinant rather than a peripheral modifier, this model introduces a novel paradigm for precision cardiovascular prevention. Beyond acknowledging psychosocial risk, the model’s novelty lies in specifying the lipid-specific crosstalk with stress biology—for example, stress-triggered NLRP3 inflammasome activation and oxidative stress that converge on endothelial dysfunction to amplify lipid-driven atherothrombosis [19,24]. This mechanistic emphasis distinguishes the framework as a biological amplifier model rather than a contextual reframing.

This Perspective aims to showcase how recent literature from the past three years has deepened our understanding of the interplay between emotional stress and cardiovascular risk, revealing that emotional states such as anger, grief, and chronic anxiety are not merely psychosocial correlates but biologically active drivers of atherosclerosis and acute events. It further seeks to critically appraise the clinical performance gap that persists within lipid-centred approaches, highlighting how emotional stress represents a modifiable yet consistently under-targeted factor in cardiovascular prevention. Finally, it emphasizes future directions by outlining how emerging emotion-sensing technologies, integrated psychosocial–lipid risk stratification frameworks, and digitally enabled therapeutics have the potential to converge into a new paradigm of precision, emotion-aware cardiovascular prevention.

This integration has transformative potential—to shift cardiovascular care from a purely biochemical model toward a biopsychocardiology paradigm that truly reflects contemporary scientific understanding and human experience.

Because this is a Perspective rather than a systematic review, we conducted a targeted scoping search of PubMed/MEDLINE, Embase, and Cochrane Central (Jan 2020–Sept 2025; last update Sept 2025) using combinations of: residual risk, LDL, apoB, triglycerides, remnant cholesterol, psychosocial stress, MSIMI, Takotsubo, microvascular dysfunction, NLRP3, endothelial dysfunction, β-blocker titration, wearables, HRV, JITAI, CBT, MBSR, Mediterranean diet, fermented foods, microbiome, SCFAs, nutritional psychiatry. We prioritized randomized trials, meta-analyses, large cohorts, and 2023–2025 reviews, but also included landmark older trials for context (e.g., CANTOS, COLCOT, LoDoCo2, PREDIMED) [25,26,27,28]. Because this is not a formal systematic review, risks of selection and publication bias remain; we therefore explicitly qualify clinical statements and call for MACE-powered RCTs of emotion-targeted care.

## 2. The Traditional Lipid-Centric Paradigm

For decades, the management of atherosclerotic cardiovascular disease (ASCVD) has been anchored upon a lipid-centric paradigm—chiefly targeting low-density lipoprotein cholesterol (LDL-C) reduction through statins, PCSK9 inhibitors, and adjunctive agents. Recent systematic analyses reaffirm that more intense LDL-C lowering is consistently associated with incremental reductions in cardiovascular events, validating the principle that “lower is better.” Novel agents, including RNA-targeted therapies and siRNA molecules, further extend this paradigm’s therapeutic reach. For instance, inclisiran—a PCSK9-targeted siRNA—has been widely implemented in several regions and offers a biannual dosing schedule, enhancing LDL-C control with high adherence potential [29,30].

Yet despite these therapeutic advances, residual cardiovascular risk remains a persistent challenge. Insights from recent trials such as FOURIER and ODYSSEY OUTCOMES reveal that even when LDL-C is aggressively lowered—down to ~30 mg/dL—approximately 9–10% of patients continue to experience major adverse cardiovascular events (MACE) within 2–3 years [31]. This persistent residual risk underscores that lipid parameters alone cannot account for all cardiovascular events. We propose that emotional stress is a key contributor to this unexplained risk. Rather than acting as a parallel pathway, stress biologically amplifies lipid-driven pathology through endothelial dysfunction, autonomic imbalance, and inflammatory activation. In this way, emotional stress fills a critical gap left by lipid-centric models and should be recognized as a co-equal determinant of cardiovascular outcomes. Beyond acting as an independent pathway, emotional stress also modifies lipid-related risk markers, further integrating the two domains. Chronic psychosocial stress and cortisol dysregulation are associated with hypertriglyceridemia and elevated remnant cholesterol—both of which independently increase ASCVD risk [32,33]. Experimental studies demonstrate that stress-induced activation of the sympathetic nervous system and HPA axis alters hepatic lipid metabolism, increases lipoprotein oxidation, and promotes the accumulation of triglyceride-rich particles [32]. These findings suggest that stress not only overlaps with traditional lipid pathways but also potentiates them, reinforcing the biological integration of emotional stress and lipid biology. Recent outcome data from triglyceride-lowering interventions further highlight the limitations of lipid-centric paradigms. In the REDUCE-IT trial, addition of icosapent ethyl (4 g/day) to statin therapy in patients with elevated triglycerides achieved a ~25% relative reduction in major adverse cardiovascular events compared to placebo (HR ≈ 0.75) [34,35]. Notably, benefits extended across strata of well-controlled LDL-C in post hoc analyses. By contrast, in the PROMINENT trial, pemafibrate substantially lowered triglyceride levels (~26%) but failed to reduce cardiovascular events (HR~1.03, *p* = 0.67) in patients with type 2 diabetes and hypertriglyceridemia [36]. Taken together, these findings illustrate that even robust triglyceride-lowering cannot by itself eliminate residual risk, underscoring the complexity of pathways beyond LDL-C and the importance of non-lipid drivers—including emotional stress—that modulate vascular biology and outcomes.

Emerging data now define the roles of non-LDL lipoproteins and biomarkers such as apoB, non-HDL cholesterol, and remnant cholesterol in capturing residual risk. A 2024 study found that higher non-HDL cholesterol levels strongly correlated with incident myocardial infarction and ASCVD—even in individuals with well-controlled LDL-C—suggesting that traditional LDL-centric thresholds may underestimate true risk [5,31,37,38,39,40,41,42]. Additionally, population-based analyses emphasize that remnant cholesterol—cholesterol carried by triglyceride-rich lipoprotein remnants—contributes substantially to ASCVD risk independently of LDL-C and is associated with systemic inflammation and heightened cardiovascular events [38].

Operationalizing emotion-aware prevention in routine care is feasible with validated tools and emerging sensors. Brief questionnaires such as the Perceived Stress Scale (PSS-10) and PHQ-9 can be implemented during intake or cardiac rehab and scored in minutes [43]. For physiologic assessment, mental stress–induced ischemia (MSIMI) protocols remain the reference for objective stress reactivity in cardiology [44]. In parallel, wearables measuring HRV (ECG/PPG), electrodermal activity, respiration/IMU, and even seismocardiography (SCG) can detect stress signatures in daily life; recent datasets and reviews show improving accuracy with multi-sensor fusion and modern ML [45,46].

Interventions with evidence. Randomized and meta-analytic data indicate that cognitive behavioral therapy (CBT), mindfulness-based stress reduction (MBSR)/digital eHealth programs, and structured exercise/cardiac rehab improve psychological outcomes and functional capacity, and in selected populations may improve clinical status [16,17,18]. Just-in-time adaptive digital interventions (JITAIs) triggered by adverse HRV dynamics have shown autonomic stabilization and superior timing efficacy in randomized and microrandomized studies, supporting translational scalability [47,48]. Pharmacologic strategies (e.g., low-dose SSRIs, sympathetic modulation) are under active study and should be viewed as adjuncts to guideline-directed lipid-lowering rather than replacements. Finally, professional guidance has begun to endorse mental-health integration in cardiovascular care, supporting implementation pathways in prevention clinics and cardiac rehab [49].

A forward-looking perspective compelled by these observations underscores two key points:

(i) Refinement of lipid targets: Therapeutic strategies should incorporate apoB or remnant cholesterol measurement into routine clinical practice—moving beyond LDL-C alone—to identify individuals at heightened risk who might benefit from additional metabolic interventions (e.g., targeting triglyceride-rich lipoproteins, ANGPTL3/4 modulation).
(ii) Integration with non-lipid risk factors: Lipid lowering, while necessary, is not sufficient. Residual risk stems from multifactorial contributors—including inflammation, emotional stress, endothelial dysfunction, and microvascular pathology—that lipid-centric interventions do not address.

From our perspective, the future of ASCVD prevention demands a paradigm shift: lipid lowering must remain a foundational intervention, but it should be embedded within a comprehensive, *precision prevention model* that includes emotional and psychosocial domains. In clinical practice, default post-MI strategies that escalate lipid-lowering—regardless of already optimal LDL-C—miss the opportunity to identify and target contributory factors such as mental stress-induced ischemia or chronic emotional dysregulation.

Thus, the next generation of cardiovascular prevention must evolve to embrace a holistic framework—combining lipid-centric therapies with emotional stress assessment and management, anti-inflammatory strategies, and digital tools capable of real-time multimodal risk monitoring. This integrative approach promises to narrow the gap between residual risk and optimal cardiovascular health.

Beyond lipids, diet and the gut–microbiome–brain axis represent modifiable mediators influencing inflammation and stress reactivity; we address these pragmatic levers in Section 5 [28,50,51].

## 3. Emotional Stress: The Underappreciated Culprit

Increasing evidence has elevated **emotional stress**—both acute psychosocial triggers (e.g., anger, grief, financial strain) and chronic stressors (e.g., anxiety, caregiving)—to a status approaching that of traditional cardiovascular risk factors. Over the last three years, mechanistic studies and clinical investigations substantiate the biological plausibility of emotional stress as both a chronic driver of atherosclerosis and an acute precipitant of cardiovascular events.

**Mental stress-induced myocardial ischemia (MSIMI)** has been extensively studied as a paradigm of how emotional triggers directly impair myocardial perfusion. A 2022 review underscores that MSIMI is often silent, occurs independently of the severity of coronary artery disease, and is associated with a twofold increase in major adverse cardiovascular events among patients with known ASCVD [52]. Recent research further shows that women—particularly younger post-MI survivors—are disproportionately affected, with depression and anxiety identified as potent predictors of MSIMI [44,53,54,55]. A 2024 mechanistic study involving women with angina and no obstructive coronary arteries (ANOCA) elucidated how mental stress impairs myocardial blood flow via microvascular dysfunction, highlighting the interaction between emotional triggers and coronary microcirculation [54,56,57]. Estrogen modulates endothelial function and autonomic tone; perimenopausal/menopausal decline may heighten vulnerability to mental-stress ischemia. Women also show higher rates of coronary microvascular dysfunction and distinct autonomic patterns that amplify stress reactivity, with implications for ANOCA phenotypes [54,58,59,60]. These considerations support sex-aware screening (e.g., targeted MSIMI, microvascular assessment) and prioritize emotion-targeted strategies in women at risk.

At the mechanistic level, emotional stress exerts pathobiological effects through multiple converging pathways: neuroendocrine activation (elevated catecholamines, cortisol), inflammatory activation (e.g., IL-6, CRP), endothelial dysfunction, and autonomic imbalance. A 2023 narrative review synthesizes these pathways, noting that psychosocial stress yields vascular injury comparable in magnitude to conventional risk factors [52]. Similarly, cross-sectional data in breast cancer survivors link greater psychosocial distress with elevated inflammatory markers (CRP, IL-6, ICAM-1) and reduced endothelial function, reinforcing the vascular consequences of sustained emotional strain [61].

We explicitly distinguish three modes by which emotional stress contributes to ASCVD: (i) mediated pathways, whereby sustained cortisol elevation promotes central adiposity and dyslipidemia—including higher triglycerides and remnant particles—thereby raising risk through conventional factors; (ii) additive effects, wherein sympathetic surges and autonomic imbalance precipitate endothelial dysfunction and microvascular constriction independent of traditional risk factors; and (iii) synergistic crosstalk, in which stress-triggered inflammation/oxidative stress (e.g., NLRP3 signaling) accelerates lipid-driven atherothrombosis. These mechanisms have been synthesized in depression-related vascular biology with glucocorticoid resistance and endothelial activation [62], experimentally linked to plaque destabilization under acute stress [56], and conceptually integrated via inflammasome-atherosclerosis pathways [24].

Emerging molecular insights further suggest that endothelial-glucocorticoid signaling dysfunction in chronic depression may exacerbate vascular pathology, including pro-inflammatory endothelial responses—an underexplored mechanistic contributor to stress-related cardiovascular disease [62].

Collectively, these developments confirm that emotional stress is not merely a comorbidity but an active contributor to ASCVD pathogenesis. From our perspective, this recognition necessitates a profound shift in clinical practice: emotional stress must be intentionally screened, monitored, and managed in cardiovascular patients—not as an afterthought, but as a central component of risk mitigation. Future preventive strategies should integrate *emotion-sensitive diagnostics* (e.g., MSIMI testing, stress symptom scales), leverage wearable biosensors to detect acute stress surges, and employ behavioral and pharmacologic interventions tailored to attenuate stress response mechanisms. This reframing aligns with a *biopsychocardiological* model—one that fully acknowledges emotional stress as a modifiable, mechanistically relevant, and clinically actionable driver of cardiovascular risk. Operational details for stress screening/monitoring and intervention pathways are summarized in Section 2 and developed further in Section 5; here we focus on mechanisms and quantitative risk context. Most links between emotional stress and ASCVD are associative, supported by large cohorts and MSIMI literature. To avoid overstating the case, we now distinguish causal signals from interventional work that target stress directly and demonstrate proximal cardiovascular benefits: (i) an open-label randomized controlled trial in Takotsubo syndrome (BREAKOUT) showed that CBT or structured exercise (vs. usual care) significantly improved 6-minute walk distance and VO_2_max and enhanced cardiac energetic/functional recovery [63]; (ii) a laboratory RCT demonstrated that experimentally induced anger acutely impairs endothelial function, establishing a direct causal pathway from negative emotion to vascular injury [11]; (iii) meta-analyses of digital/eHealth stress-management programs report significant improvements in depression/anxiety/stress among CVD patients, supporting scalable delivery [16,18]; and (iv) microrandomized JITAI trials triggered by adverse HRV dynamics show autonomic stabilization and superior timing efficacy [47]; related JAMA Network Open work confirms the causal leverage of timing in behavior-change RCTs [48]. We also note that cardiac rehabilitation consistently improves HRQoL and psychological outcomes in CVD [17]. Taken together, these trials support plausible causal pathways and proximal cardiovascular benefits of stress-targeting interventions; however, adequately powered outcome trials for hard endpoints (MACE) remain limited—hence our proposal for EMOTION-MI.

To complement the mechanistic and cohort evidence, we provide effect-size anchors so clinicians can gauge the magnitude of stress-related risk against conventional factors. Specifically, we summarize relative risks from trigger/case-crossover analyses and population-attributable risk (PAR) estimates from global comparative risk assessments, noting expected variability by age, geography, and exposure prevalence [64,65]. Case-crossover and meta-analytic data show that acute emotional stress (anger/emotional upset) is associated with an ~2-fold short-term increase in acute myocardial infarction (AMI) risk (INTERHEART case-crossover; OR ~2.0) [64]. Findings are consistent across systematic work on anger as a trigger for acute cardiovascular events [66,67]. At the population level, the population attributable risk (PAR) of acute emotional stress for acute coronary events is commonly estimated around ~5–10% (range varies by method and exposure prevalence; consistent with trigger literature above). For comparison with *conventional* factors, hypertension accounts for roughly ~20–25% of vascular mortality, and diabetes for ~10–15% of ASCVD burden at the population level, based on large collaborative and GBD analyses [65,68].

To further illustrate the role of emotional stress compared with traditional cardiovascular risk factors, Table 1 summarizes their chronic and acute mechanisms, relative risk estimates, and approximate population attributable risk (PAR) where available [9,31,55,65,69,70,71].

## 4. Mechanistic Insights: The Emotion–Lipid Synergy Model

A pivotal insight emerging over the past three years is that acute emotional stress does not merely trigger symptoms—it mechanistically potentiates lipid-driven processes, accelerating both atherogenesis and thrombosis through a coordinated cascade of neuroendocrine, immunologic, and vascular effects.

Emotion-induced activation of the sympathetic nervous system and the hypothalamic–pituitary–adrenal (HPA) axis elevates catecholamines and cortisol, which rapidly exacerbate endothelial dysfunction and hemodynamic stress. These neurohumoral changes increase shear stress and microvascular constriction, impairing myocardial perfusion. Simultaneously, a pro-inflammatory cascade activates the NLRP3 inflammasome, amplifying the secretion of IL-1β and IL-18, which, in concert with oxidative stress, undermines vascular homeostasis and promotes plaque instability. Beyond endothelial dysfunction, stress hormones and redox biology modify lipid handling and lipoprotein quality. Acute mental stress accelerates vascular inflammation and plaque destabilization in experimental atherosclerosis, implicating catecholamine surges and shear stress in oxidized LDL accrual and macrophage uptake (*foam-cell* formation) [56]. Chronic cortisol dysregulation is associated with hypertriglyceridemia and remnant cholesterol via hepatic lipid metabolism changes [33,38]. Together, these data indicate that stress alters lipid metabolism and lipoprotein atherogenicity directly, in addition to its inflammatory and vasomotor effects. Recent reviews reinforce this pathophysiologic interconnection, describing how NLRP3 acts as a fulcrum linking inflammatory signals with endothelial pathology in atherosclerosis—including oxidative stress and mitochondrial dysfunction—thus bridging lipid-mediated injury with emotional stress pathways [19,20,21,22,23,24].

At the level of myocardial perfusion, MSIMI exemplifies the immediate adverse impact of emotional stress even when lipid levels are well controlled. MSIMI often occurs at lower levels of exertion, is frequently clinically silent, and is driven by neurovascular dysregulation rather than fixed coronary obstruction [55,72,73]. Women, especially those post-myocardial infarction, display heightened susceptibility to MSIMI and microvascular dysfunction—a disparity that recent reviews suggest may be partly rooted in sex-specific endothelial sensitivity and autonomic patterns [58,60,74,75,76,77,78,79]. Estrogen favors endothelial NO bioavailability and autonomic balance; its perimenopausal/postmenopausal decline may heighten stress-induced vasoconstriction. Women also exhibit greater prevalence of coronary microvascular dysfunction and sex-specific autonomic patterns, which together amplify stress reactivity and MSIMI risk [54,58,59].

*Takotsubo syndrome*—the classic “broken heart” cardiomyopathy—is perhaps the most striking clinical embodiment of emotionally driven myocardial injury. Contemporary studies confirm that emotional stress precipitates massive catecholamine surges, inducing oxidative stress and endothelial dysfunction. Key molecular players such as heme oxygenase-1 (HO-1) are upregulated in affected myocardium, while α- and β-adrenergic pathways mediate oxidative injury and cardiomyocyte stunning. Oxidative stress thereby emerges as a critical substrate for transient left ventricular dysfunction in Takotsubo syndrome [21,22,23].

At the population scale, persistent cortisol exposure remodels metabolism—elevating VLDL/TG, promoting central adiposity, and favoring triglyceride-rich lipoproteins that track with residual risk; it also accelerates endothelial senescence and maladaptive plaque remodeling, sustaining a pro-atherogenic milieu [12,62]. These chronic processes complement the acute-trigger phenomena (MSIMI, Takotsubo), highlighting distinct time-scales through which stress increases ASCVD risk.

Collectively, these mechanisms underscore the **Emotion–Lipid Synergy Model**: emotional stress amplifies the pathophysiologic consequences of lipid-mediated vulnerability—via inflammatory, endothelial, and neurohumoral convergence—thus inflating residual risk even when lipids are adequately managed.

Looking ahead, several avenues appear particularly promising. One important target is the NLRP3 inflammasome, which occupies a dual role in both inflammation and lipid-driven atherogenesis; novel therapeutics aimed at inhibiting its activation, whether through small-molecule inhibitors or other modulators, may substantially reduce the synergistic burden imposed by emotional stress and dyslipidemia. Equally critical is the development of sex-specific risk stratification, as accumulating evidence shows that women are disproportionately vulnerable to stress-related mechanisms such as MSIMI and Takotsubo cardiomyopathy. Incorporating microvascular function testing and stress reactivity assessments into standard practice could help tailor prevention strategies more precisely across sexes. At the same time, integrated diagnostic frameworks that combine circulating biomarkers (e.g., IL-1β, IL-18, HO-1), advanced imaging modalities such as microvascular perfusion reserve, and wearable-derived physiological signals hold potential for real-time detection of synergistic emotional–lipid risk. Finally, the future of cardiovascular prevention must move toward multi-modal interventions that transcend lipid-lowering alone. This includes pharmacological strategies—ranging from anti-inflammatory and autonomic modulators to cytokine-targeted therapies—as well as behavioral and digital approaches designed to improve emotion regulation and stress resilience. Together, these integrative pathways offer the possibility of interrupting the vicious feedback loops through which emotional stress magnifies lipid-driven cardiovascular risk, pointing toward a more complete and truly personalized model of prevention. Circulating IL-1β and IL-18 index inflammasome activity relevant to stress–lipid crosstalk; heme oxygenase-1 (HO-1) is upregulated in stress cardiomyopathy and reflects oxidative stress [19]. For imaging, coronary flow reserve (PET/Doppler) and microvascular perfusion reserve (CMR/PET) are practical markers of stress-related microvascular dysfunction and are increasingly available in tertiary centers [54]. CBT and structured exercise improved 6 min walk distance and VO_2_max in stress-triggered cardiomyopathy (BREAKOUT RCT) [63]. A laboratory RCT established that experimentally induced anger acutely impairs endothelial function—a direct causal link between negative emotion and vascular injury [11]. eHealth/MBSR-style programs in CVD populations show clinically meaningful improvements in depression, anxiety and stress in meta-analyses [16,18]. JITAI programs triggered by adverse HRV dynamics demonstrate autonomic stabilization in microrandomized trials and the causal leverage of timing in an RCT [18,48]. These strategies should complement (not replace) guideline-directed lipid lowering, and align with ESC 2025 recommendations to integrate mental health into CV care [49]. Our mechanistic model nominates innate-immune signaling (IL-1→IL-6→CRP) and inflammasome/oxidative–endothelial crosstalk as tractable nodes. Clinically, IL-1β inhibition (canakinumab) reduced recurrent events independent of lipids (CANTOS) [25], and low-dose colchicine lowered MACE in chronic CAD and post-MI populations (LoDoCo2, COLCOT) [27]. Upstream IL-6 ligand inhibition (ziltivekimab) robustly reduced inflammatory biomarkers in RESCUE (phase 2) and is being tested for hard outcomes in ZEUS (phase 3) [80]. These pharmacologic levers complement behavioral/digital stress modulation and align with our Emotion–Lipid Synergy pathways.

**Figure 1:**
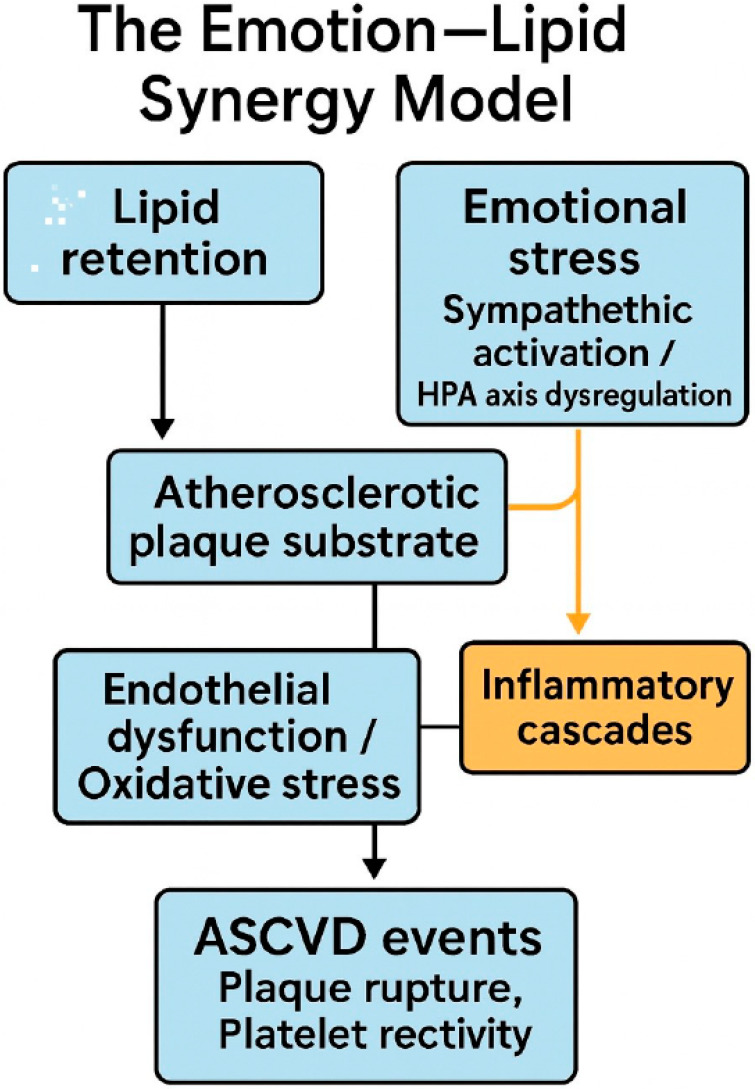
The Emotion–Lipid Synergy Model. Lipid retention initiates atherosclerotic plaque formation, providing the biological substrate for disease. Emotional stress—including anger, grief, and chronic psychosocial strain—triggers sympathetic activation, HPA axis dysregulation, and inflammatory cascades. These processes converge on endothelial dysfunction, oxidative stress, and heightened platelet reactivity, collectively amplifying the risk of plaque rupture and acute cardiovascular events, even in patients with well-controlled LDL-C.

A schematic representation of the Emotion–Lipid Synergy Model is shown in Figure 1. This diagram illustrates how lipid retention creates the atherogenic substrate, while emotional stress—through neuroendocrine activation and inflammatory signaling—exacerbates endothelial dysfunction, promotes thrombosis, and accelerates ASCVD progression.

In summary, the Emotion–Lipid Synergy Model reframes emotional stress from a peripheral psychosocial variable into an integrally linked pathobiological force. By elucidating these mechanisms, we illuminate strategic avenues for truly integrative, emotion-aware precision cardiovascular prevention.

## 5. Clinical and Therapeutic Implications

The convergence of emerging evidence underscores the vital need to bridge the gap between traditional lipid management and the overlooked domain of emotional stress—both as a driver of residual cardiovascular risk and as an actionable therapeutic target. Notably, even at very low LDL-C, patients remain at risk through *stress-mediated* inflammatory, autonomic, and microvascular pathways—precisely the layer of residual risk operationalized by the Emotion–Lipid Synergy Model [31,81]. Recent clinical and meta-analytic studies robustly demonstrate that psychological and behavioral interventions yield meaningful benefits for cardiovascular patients.

In an RCT of stress-triggered cardiomyopathy, CBT or structured exercise (vs usual care) produced clinically meaningful functional recovery, with consistent gains in 6 min walk distance and cardiopulmonary fitness, supporting the role of targeted stress interventions in rehabilitation [63]. Given the disproportionate burden of MSIMI and ANOCA in women, emotion-targeted strategies (stress reactivity monitoring, CBT, mindfulness, digital self-regulation) may yield greater clinical benefit in this group; mechanistic susceptibility involves microvascular dysfunction and sex-specific autonomic patterns [54,58]. In patients with chronic cardiovascular disease, digital (eHealth/mHealth) stress-management interventions are also gaining traction. A comprehensive systematic review and meta-analysis of 35 randomized trials concluded that eHealth-based psychological interventions reduce depression, anxiety, and stress in patients with coronary and other heart conditions—a key finding that underscores the scalability and real-world applicability of digital therapeutics in this clinical context [16,18]. Across trials, pooled effects are in the *small-to-moderate* range (typical SMD ≈ 0.3–0.5 for depression/anxiety/stress), which corresponds to an approximate NNT of ~7–10 [16,17,18]. Another meta-analysis reinforced these findings, revealing that eHealth stress interventions significantly improve psychological health parameters such as depression scores and mental health–related quality of life among CVD patients [16,17,18,49,82,83,84,85,86,87,88].

Beyond symptom improvement, stress reactivity itself may portend cardiovascular risk. A novel risk score derived from cardiovascular reactivity to mental stress predicted adverse cardiovascular outcomes independently of traditional risk factors, emphasizing that dynamic stress assessments could meaningfully refine risk stratification models [86]. In practice, these composite scores incorporate: (i) blood-pressure rise under standardized mental stress, (ii) HRV reductions (autonomic imbalance), and (iii) endothelial reactivity during/after stress (e.g., brachial FMD or peripheral arterial tonometry), each associated with prognosis in CAD [86].

Moreover, a recent clinical consensus statement from the European Society of Cardiology (ESC) has begun to acknowledge the interplay between mental health and cardiovascular risk, especially in individuals without known CVD, marking a shift toward integrating psychological dimensions in risk assessment frameworks [49].

From a rehabilitation standpoint, psychological distress remains under-screened. Although cardiac rehabilitation programs are now recognized as a venue for detecting and addressing psychosocial risk, emotional screening tools are still underutilized, highlighting a significant implementation gap that could be readily addressed to improve patient-centered care [89,90,91].

In our expert judgment, these findings together signal a transformative opportunity: to reconceive cardiovascular care as truly biopsychocardiological, where emotional health is not only assessed but actively treated in parallel with pharmacotherapy and lifestyle modification. Clinicians should shift toward embedding psychological screening (e.g., PHQ-9, GAD-7, stress reactivity tests) routinely in post-MI and preventive care settings, especially among populations such as stress-sensitive women, those with manifest microvascular dysfunction, or survivors of takotsubo syndrome.

On the therapeutic front, integrated models might include:

(i) Emotion-targeted interventions: Combining pharmacologic options (e.g., low-dose SSRIs, centrally acting agents) with structured behavioral therapies such as CBT or mindfulness-based stress reduction (MBSR).
(ii) Digital therapeutics: Deploying eHealth and mHealth platforms capable of delivering CBT, relaxation, or emotion-regulation modules, particularly suited for long-term adherence and monitoring.
(iii) Exercise integration: Leveraging structured recovery programs—not only to improve physical conditioning post-MI or takotsubo—but also as psychological therapy, capitalizing on the mutually reinforcing benefits of physical activity and emotional regulation.
(iv) *Dietary patterns, microbiome, and emotion-aware prevention*: Diet is a tractable mediator at the intersection of lipid metabolism, inflammation, and stress physiology. In primary prevention, Mediterranean-style eating reduces major cardiovascular events (PREDIMED; randomized re-analysis) [28]. At the immune–microbiome interface, a randomized diet trial in healthy adults showed that fermented foods (vs high-fiber alone) increased microbiome diversity and decreased inflammatory markers over 10 weeks [50]. Preclinical and translational data support the microbiota–gut–brain axis and short-chain fatty acids (SCFAs) as mediators of stress and neuroimmune signaling, nominating diet as a pathway to alter stress biology [51,92]. In nutritional psychiatry, the SMILES randomized trial demonstrated that a structured diet intervention improved depressive symptoms with an NNT ≈ 4 for remission [93]. Taken together, diet can be integrated with lipid-lowering and emotion-targeted strategies to reduce residual risk through apoB/LDL pathways, inflammatory tone, and stress reactivity.

While more trials are needed to test how combined lipid-emotional interventions impact hard cardiovascular endpoints, the existing data support an immediate step: incorporating validated psychological and digital tools into routine CVD management. This move not only aligns with the precision prevention ethos but also addresses a major unmet need evidenced across recent clinical literature.

Recommendations herein are adjunctive and should be framed as “consider” or “reasonable in selected patients” under shared decision-making until MACE-powered RCTs of stress-targeted care report. Notably, anti-inflammatory strategies aligned to our pathway do reduce hard outcomes (e.g., canakinumab 150 mg q3mo; HR~0.85 for primary endpoint in CANTOS; colchicine 0.5 mg daily reduced events in COLCOT and LoDoCo2) [25,26,27,28]. By analogy, EMOTION-MI is designed to test whether adding emotion-aware care to guideline lipid management reduces MACE.

## 6. Digital Health and Precision Prevention

In the past three years, digital biomarkers and adaptive software have matured enough to make emotion-aware cardiovascular prevention technically feasible. On the sensing side, modern wearables now capture multi-modal physiology—ECG/PPG for beat-to-beat variability, electrodermal activity and skin temperature for sympathetic arousal, respiration from chest/IMU signals, and even seismocardiography (SCG) from chest-worn accelerometers—to infer stress and affective state continuously in daily life. New open datasets (e.g., EmoWear) explicitly benchmark these signals for emotion recognition across valence–arousal space, enabling head-to-head comparisons of ECG, blood-volume pulse (PPG), respiration, EDA, and SCG under controlled emotion elicitation; notably, SCG plus accelerometry-derived respiration performs on par with ECG/PPG while requiring only a small chest sensor, improving wearability for ambulatory monitoring [45]. Systematic reviews from 2024–2025 converge on the same message: stress and emotion can be detected from consumer-grade wearables with increasing robustness when multi-sensor fusion and modern ML are used, although real-world generalization and motion-artifact resilience remain the rate-limiting steps [46,94,95]. Importantly, these digital signatures (falling HRV, electrodermal surges, tachypnea) reflect catecholamine surges, microvascular constriction, and autonomic lability—the same stress-mediated processes that amplify lipid-driven ASCVD risk in the Emotion–Lipid Synergy Model. In our view, cardiology should now treat these signals as first-class inputs—not curiosities—because they capture the very phenomena (sympathetic bursts, microvascular constriction, hyperventilation, autonomic lability) that mediate mental-stress ischemia and trigger events in vulnerable patients.

A second enabling pillar is the data/AI layer. Labeling “ground-truth” emotions is expensive and error-prone, so the field has pivoted to self-supervised learning (SSL) on large volumes of unlabeled biosignals to pretrain models that later fine-tune with small labeled sets. Recent work shows SSL markedly improves emotion recognition from biosignals (demonstrated in EEG and readily extensible to ECG/PPG/EDA), precisely because it exploits invariant structure (rhythms, morphology, co-modulation) without curated labels [96]. In parallel, 2025 overviews of open multimodal stress/emotion datasets catalog the sensing channels, protocols, and annotation schemes available for reproducible research—an essential substrate for rigorous model evaluation and federated, privacy-preserving training [97]. Our assessment: the near-term wins will come from foundation models for physiology (i.e., large pretrained AI models built from months of wearable biosignal data, which can then be customized for each patient), adapted on-device to each patient to capture idiosyncratic stress signatures while keeping protected health data local.

Crucially, sensing and modeling only create value when they change what patients do in the moment. That is the remit of Just-in-Time Adaptive Interventions (JITAIs)—micro-interventions (e.g., 60–120 s of paced breathing, attentional refocusing, cognitive reappraisal, or a brief CBT skill) delivered exactly when physiology and context indicate rising risk. The last two years brought encouraging evidence: (i) microrandomized trials demonstrate that smartphone-prompted 0.1 Hz breathing or brief attention-focus exercises reduce perceived stress and stabilize cardiac autonomic function when triggered by adverse HRV dynamics, versus random delivery; (ii) a JAMA Network Open RCT showed that a tailored, JITAI-driven digital program outperformed standard digital care in behavior-change efficacy (in that case, smoking cessation), reinforcing the causal leverage of timing and personalization; (iii) JITAI feasibility and tailoring-variable studies now identify which context and sensor features best predict momentary receptivity and adherence—critical for cardiology use where alert fatigue is dangerous [47,98]. Complementary evidence from remote cardiac rehabilitation indicates that wearable-guided, coach-augmented home programs improve exercise capacity—an implementation channel through which emotion-aware prompts can be integrated into standard care [99]. Recent narrative/systematic reviews in 2024–2025 also map the JITAI design space and highlight current gaps (decision rules under-specified, limited use of passive sensing for triggering), providing a concrete agenda for higher-quality, cardiology-specific trials [100,101,102]. Widespread adoption of digital stress-monitoring requires clear regulatory pathways (e.g., FDA/CE), interoperability with EHRs (standards-based ingestion of wearable summaries), and reimbursement models for clinician time and digital therapeutics. Governance should include privacy-preserving, on-device inference with minimal data transfer, consistent with emerging consensus on integrating mental-health dimensions into CV care [49].

Our forward view for cardiology

(i) From numbers to states: Continuous lipid metrics are static; emotional state is dynamic. In patients with established CAD (or post-MI), we advocate pairing apoB/LDL targets with state-space monitoring (HRV, EDA, breathing, SCG) to detect “high-risk emotional states” that can precipitate ischemia or arrhythmia, then responding in minutes—not months—with a micro-intervention (paced breathing, brief CBT, or even short-acting pharmacologic modulation) delivered as a JITAI [47].
(ii) Precision triggers, layered treatments: Use patient-specific physiological fingerprints (e.g., a characteristic HRV drop + EDA surge) as digital triggers that titrate the intensity of the intervention: from a subtle haptic cue to breathe, up to a coached CBT module, and, for selected patients, protocolized β-blocker up-titration or anti-inflammatory strategies if repeated stress-peaks correlate with symptoms or biomarkers. (Trials should test these stepped algorithms explicitly).
(iii) On-device, privacy-preserving AI: Deploy SSL-pretrained models on the phone/watch to keep raw data local, fine-tune to the individual, and stream only low-dimensional risk scores to the clinic (i.e., brief, clinically interpretable indices such as a stress-reactivity score derived from HRV/EDA)—minimizing privacy/latency issues while maximizing clinical actionability [96].
(iv) Equity by design: Because stress burden is socially patterned, digital prevention must not widen disparities. Plan for device access (loaner wearables, subsidized programs), connectivity (offline/sms pathways where broadband is limited), and digital literacy (multilingual, simple UI). Co-design with communities at highest risk and evaluate in pragmatic trials that oversample under-resourced settings [103,104,105,106,107,108,109]. Test feasibility alongside outcomes to ensure inclusive benefit, not just technical success [101,110].

If we execute on this roadmap, digital tools will finally let cardiovascular teams see and treat the emotional physiology that loads the dice for acute events—closing a major slice of residual risk that lipid-only strategies cannot reach.

## 7. Equity & Social Determinants

A refined understanding of residual cardiovascular risk must account for the profound influence of social determinants of health (SDoH)—especially socioeconomic status, caregiving burdens, and structural inequities—that disproportionately amplify the emotional stress burden among women, ethnic minorities, and economically disadvantaged populations. Throughout this section, we use a single motif—chronic stress as a socially patterned amplifier of residual risk—to link socioeconomic position, race/ethnicity, racism-related stress, and caregiving burden to persistent cardiovascular disparities [13,104,105,111,112].

Recent evidence reaffirms that lower socioeconomic status is strongly associated with elevated cardiovascular risk, mediated both directly—through limited healthcare access and poor living conditions—and indirectly via emotional stress, depression, and engagement in unhealthy behaviors. A 2025 narrative review highlights how SES shapes both psychological and physical health outcomes, with gender-specific vulnerabilities magnifying risk in women of lower-income backgrounds [103,104,105,106,107,108,109].

Racial and ethnic disparities further compound this risk. A 2024 population-based study in the U.S. reported that Black and Hispanic patients with cardiovascular disease are far more likely than their White counterparts to report poorer perceived health, even after adjusting for income, education, and comorbidities [113]. Structural factors such as discrimination and neighborhood deprivation also emerge as pivotal. A JAMA Network Open analysis showed that neighborhood stressors and social environment differences largely attenuate racial disparities in cardiovascular health outcomes—underscoring that place and context matter deeply [13,104,105,111,112]. In preventive cardiology and cardiac rehab, extend stress screening beyond depression/anxiety scales to include brief, structured questions on caregiving burden, financial strain, and experiences of discrimination—domains that track with adverse outcomes but are often missed in routine intake [91].

On a psychosocial axis, racism-related stress acts as a chronic, toxic burden. A 2025 commentary synthesizes how repeated exposure to discrimination—interpersonal and institutional—promotes allostatic load, inflammation, and autonomic dysregulation, ultimately increasing ASCVD risk [114]. Corroborating this, a 2024 behavioral study linked experiences of racial or ethnic discrimination to immediate elevations in cardiovascular reactivity, reflecting acute physiological stress responses that cumulatively impair cardiovascular resilience [107]. These findings echo broader conceptual models, such as the weathering hypothesis, which attributes accelerated health decline among Black women to the cumulative wear-and-tear of chronic stressors, including racism and socioeconomic disadvantage [115].

Furthermore, caregiving stress—especially among individuals of lower SES—intensifies emotional burden and downstream health risks. Analogous research in oncology caregiving shows that financial strain, reduced workforce participation, and educational disparities heighten psychological distress, with potential parallels in cardiovascular caregiving contexts [116,117]. Care partners of post-MI and heart failure patients frequently report high emotional strain, reduced workforce participation, and elevated depressive symptoms—patterns associated with increased cardiovascular risk in caregivers themselves. Recognizing and addressing caregiver stress (education, respite, social support, digital stress-management) should be part of comprehensive secondary prevention planning [116].

### From Perspective to Action

Moving forward, a biopsychocardiological framework that centers on health equity must guide both research and practice. First, risk stratification models should integrate SES and psychosocial stressors alongside traditional risk factors, recognizing that emotional stress mediates a significant share of residual risk and is socially patterned. Second, culturally informed interventions are essential: stress reduction and digital therapeutic tools (e.g., JITAIs) should be co-designed with the lived experiences of marginalized communities in mind—ensuring relevance, accessibility, and trust. Third, healthcare systems must commit to structural reforms, such as enhancing access to preventive and specialist cardiovascular care in deprived areas and addressing referral biases—especially for heart conditions such as aortic stenosis, where women and racial minorities face documented treatment disparities [118]. To translate equity aims into practice, systems should prioritize insurance coverage for digital therapeutics and stress-management interventions, funding for community-based cardiac rehabilitation, and reimbursement for psychosocial screening in cardiology clinics. These actions align with emerging professional guidance on integrating mental-health dimensions into cardiovascular care [49].

Our personal assessment is clear: without embedding equity at the core, the Emotion–Lipid Synergy model risks deepening disparities. It is incumbent upon clinicians, researchers, and policy leaders to dismantle structural inequities that amplify emotional stress—and to deliver emotion-integrated prevention in an inclusive, justice-oriented manner.

## 8. Future Directions and Research

### 8.1. Flagship Outcomes Trial: EMOTION-MI

We propose a pragmatic, multicenter, EMOTION-MI trial to test whether adding emotion-aware care to guideline-directed lipid management reduces post-MI events. Adults within 6–12 weeks of type 1 MI, on high-intensity statins (±ezetimibe/PCSK9i) with apoB ≤ 80 mg/dL (to minimize confounding from undertreated lipids) would be randomized (1:1:1:1) by site to: (A) usual care; (B) wearable emotion-sensing with alerts plus just-in-time breathing prompts; (C) the same + protocolized β-blocker titration when repeated stress-reactive hemodynamic abnormalities are detected; or (D) the same + an 8-session digital CBT program targeting anger reappraisal, attentional shifting, and paced breathing. Primary endpoint: 3-year MACE (CV death, MI, stroke, unplanned revascularization). Key secondaries: mental-stress reactivity risk score (validated prognostically in 2025), endothelial function response to brief anger provocation (shown to deteriorate in RCT conditions), MSIMI prevalence/severity in a mechanistic substudy of 600 patients (including women with ANOCA/microvascular dysfunction), health-related quality of life, and cost-effectiveness. Digital triggers would be derived from HRV/PPG/SCG **+** electrodermal features, informed by recent open datasets and microrandomized JITAI trials that demonstrated superior stress reduction and autonomic stabilization when interventions are timed by adverse HRV dynamics rather than delivered randomly. To address scale and cost while preserving rigor, EMOTION-MI will consider adaptive designs—for example, SMART sequences (re-randomizing non-responders between digital/breathing, protocolized β-blocker titration, and digital CBT) and Bayesian response-adaptive randomization to concentrate allocation on arms with emerging benefit while controlling error rates. We also clarify the β-blocker titration arm: titration will be protocolized when repeated stress-reactive abnormalities are detected (e.g., reproducible stress-provoked blood-pressure/heart-rate variability changes or MSIMI on standardized tasks), whereas standard care titration remains symptom-driven and based on routine resting vitals. This ensures scientifically rigorous and clinically meaningful comparators aligned with the trial’s mechanistic intent. This design directly addresses calls from recent consensus and review papers to integrate mental health assessment within cardiology and to move from association to causal tests of emotion-targeted strategies [11,45,47,48,49,54,86].

The trial builds on three developments from the past three years: (i) consensus that psychological distress independently worsens prognosis and improves risk prediction in CHD; (ii) experimental proof that anger alone impairs endothelial function, nominating a tractable physiologic endpoint; and (iii) feasibility of wearable-triggered, just-in-time adaptive interventions (JITAIs) that outperform static digital care in randomized trials and stabilize cardiac autonomic function in microrandomized experiments. By restricting enrollment to patients already at low apoB, EMOTION-MI isolates the residual risk layer that current lipid-centric care does not reach [9,11,47,48].

### 8.2. Mechanistic Research Priorities

Two near-term priorities should sharpen causal inference and therapeutic targeting. First, map stress → endothelium and stress → inflammation pathways with standardized laboratory anger/sadness tasks linked to vascular readouts (brachial FMD or PAT) and cytokine panels (IL-6, IL-1β, CRP) at serial time points; prioritize sex-stratified analyses given demonstrated female susceptibility to MSIMI and microvascular constriction. Second, embed neuro-autonomic phenotyping (HRV, vasoconstrictive responses, respiration) within these protocols to refine the reactivity risk score and test whether improvements induced by JITAI/β-blockade track with event reduction. These directions align with 2024–2025 literature clarifying stress biology, microvascular mechanisms of MSIMI in women, and the prognostic value of composite mental-stress reactivity metrics [12,54,86]. Exploratory neuroimaging with functional near-infrared spectroscopy (fNIRS) during standardized anger/sadness tasks will quantify cortical hemodynamics/cerebrovascular reactivity, linking emotional stress to endothelial function and perfusion endpoints in ASCVD [49].

### 8.3. Precision Phenotyping and Biobanking

Create a prospective, emotion–lipid–immunology biobank that couples continuous wearable streams (ECG/PPG/SCG/EDA), ecological momentary assessments, fasting lipids (apoB), inflammatory markers, and periodic endothelial function testing. Leverage self-supervised learning on long, unlabeled physiologic time series to derive person-specific “stress fingerprints,” then link these to plaque vulnerability surrogates and outcomes. Open benchmark datasets (e.g., EmoWear) and recent reviews on multimodal stress detection provide the scaffolding to standardize signals and labels; foundation models for physiology can be fine-tuned on-device to preserve privacy and adapt to individual phenotypes. The biobank should oversample women and racially/ethnically diverse groups to ensure generalizable sex- and equity-aware classifiers [45]. To enable multi-site learning while protecting participants, we will use federated learning so models train across sites without centralizing raw data, controlled-access repositories for biosignals/biomarkers, and on-device inference for wearable analytics so only compact summaries are shared with the clinic—approaches consistent with recent stress/emotion data infrastructure guidance [97].

### 8.4. Implementation Science and Real-World Integration

To move from pilots to practice, cardiology needs scalable workflows that embed emotion metrics into routine care. Building blocks include: (i) adoption of ESC 2025 recommendations to integrate mental health into person-centered CV care; (ii) use of remote/virtual cardiac rehabilitation as a delivery channel for emotion-aware coaching and JITAIs; (iii) EHR integration of a stress reactivity score and alert thresholds; and (iv) privacy-preserving, on-device inference that transmits only low-dimensional risk flags. Because device access and digital literacy are uneven, we explicitly address older adults, rural communities, and socioeconomically disadvantaged groups: (i) deploy community device-lending/subsidy programs; (ii) provide simplified, large-font, multilingual interfaces and hands-on onboarding; (iii) design offline/SMS pathways where broadband is limited; and (iv) evaluate equity in pragmatic and cluster-randomized trials that oversample under-resourced settings. These safeguards prevent widening disparities and align with current guidance on equitable digital mental-health integration in cardiovascular prevention [11,49]. Hybrid effectiveness–implementation trials should evaluate uptake, fidelity, equity, and cost alongside clinical outcomes [49].

**Our assessment.** The field is ready to test emotion-aware precision prevention head-to-head with lipid-only models. The literature from 2023–2025 gives us the ingredients—validated distress predictors, mechanistic endothelial endpoints, wearable-triggered interventions that work when timed correctly, and professional society support for integrating mental health into CV care. The next step is disciplined, adequately powered trials and biobanks that let us learn who benefits, when, and through which biological levers—and then make those insights deployable in everyday cardiology.

## 9. Conclusions

Cardiovascular prevention has long rested on the foundation of lipid management, with statins, PCSK9 inhibitors, and emerging siRNA-based agents dramatically lowering LDL-C and apoB. However, even with optimal lipid control, residual risk persists—a clinical reality underscored by randomized trials and meta-analyses showing event rates remain significant in well-treated populations (e.g., 9–10% experiencing MACE within two to three years) [81].

In parallel, compelling evidence from the last three years substantiates the role of emotional stress as a biologically potent driver of cardiovascular risk. Psychological distress independently predicts recurrent events among patients with coronary artery disease [9], while controlled experiments demonstrate that acute emotional triggers, such as anger, directly impair endothelial function and activate inflammatory pathways [11]. Notably, women—particularly those with angina and non-obstructive coronary arteries—are disproportionately vulnerable to mental stress–induced myocardial ischemia [54], reinforcing the need for sex-conscious preventive strategies.

This evolving knowledge underpins the proposed *Emotion–Lipid Synergy Model***,** which situates emotional stress not as a peripheral factor but as a core, mechanistic amplifier of lipid-driven pathology. In our view, the future of cardiovascular prevention must evolve into a precision psychocardiology paradigm—a model that converges lipid-lowering, emotion detection via wearables and biomarkers, and stress-modulating interventions. Taken together, these insights underscore that stress and lipid biology are deeply intertwined and must be targeted together if we are to close the gap in residual cardiovascular risk.

Pragmatically, recent advances such as wearable emotion-sensing platforms capable of detecting real-time stress signatures [45], and just-in-time adaptive interventions that have demonstrated efficacy in reducing autonomic reactivity and perceived stress [47], make this vision technically feasible. The endorsement of psychosocial integration in cardiovascular care by leading cardiovascular societies—such as the 2025 ESC consensus on mental health and CVD—further solidifies the clinical imperative to act [49]. Equally important, deployment must include equity safeguards—subsidized device access, connectivity alternatives, and culturally adapted interfaces—so that digital exclusion does not widen existing cardiovascular disparities [49,103,104]. Clinicians can begin integrating structured stress assessments (e.g., PHQ-9, Perceived Stress Scale) into routine preventive care today, alongside lipid screening, as a pragmatic step toward emotion-aware cardiovascular prevention [49].

We believe that the decade ahead offers an unprecedented opportunity: by conducting trials like EMOTION-MI, pursuing mechanistic and phenotyping research, and ensuring equitable deployment of emotion-integrated care, we can finally close the gap in residual risk. While promising pathways are emerging and uncertainties remain, if we treat emotion as both a measurable and modifiable domain, we can realign cardiovascular prevention with the complex reality of human biology and resilience.

## Figures and Tables

**Table 1:** Comparison of traditional cardiovascular risk factors and emotional stress. Traditional risk factors such as hypertension, hyperlipidemia, smoking, and diabetes exert their effects predominantly through chronic vascular injury, metabolic dysregulation, and inflammatory activation, with acute triggers amplifying risk via hemodynamic surges or prothrombotic changes. Emotional stress, both chronic (e.g., sustained psychosocial strain) and acute (e.g., anger, grief), operates through sympathetic activation, hypothalamic–pituitary–adrenal axis dysregulation, and inflammatory cascades, converging on endothelial dysfunction and plaque instability. While relative risk estimates for traditional factors are well established, emerging evidence suggests that acute emotional stress may double the short-term risk of myocardial infarction, with a population attributable risk of ~5–10% for acute coronary events. ^1^ RR = Relative risk; approximate values from large meta-analyses and representative cohorts. ^2^ PAR = Population attributable risk; approximate population-level estimates.

Risk Factor	Chronic Effect Mechanism	Acute Trigger Mechanism	Estimated Relative Risk (RR) [1]	Population Attributable Risk (PAR) [2]	References
Hypertension	Sustained high arterial pressure → endothelial injury, left ventricular hypertrophy	Acute surges in BP raise shear stress and plaque rupture risk	~1.8 per 20 mm Hg increase	~20–25% in many populations	[70]
Hyperlipidemia	ApoB-containing lipoproteins drive plaque formation	Extremely high levels may precipitate plaque rupture	~1.6 per mmol/L LDL-C increase	~17–20% globally	[31]
Smoking	Chronic inflammation, oxidative stress, endothelial damage	Acute platelet activation, vasoconstriction	~2–3× vs. non-smokers	~15–25% in high-prevalence groups	[65]
Diabetes	Accelerated atherosclerosis via glycation, inflammation	Acute hyperglycemia impairs vascular function	~2× risk of MI	~10–15% in most populations	[71]
Emotional Stress	Chronic sympathetic activation, allostatic load, inflammation	Acute anger or grief triggers MSIMI and plaque rupture	RR ~2.0 for acute MI trigger	Estimated ~5–10% of acute MIs	[9,55]

**Notes:** [1] **Estimated relative risk per standard unit increase or exposure.** [2] Approximate population-attributable risk based on epidemiological data.

## Data Availability

No new data were created or analyzed in this study. Data sharing is not applicable to this article.
